# IgG4-Mediated Sclerosing Riedel Thyroiditis: A Multidisciplinary Case Study and Literature Review

**DOI:** 10.3390/ijms26167786

**Published:** 2025-08-12

**Authors:** Dumitru Ioachim, Mihai Alin Publik, Dana Terzea, Carmen Adina Cristea, Adina Mariana Ghemigian, Anda Dumitrascu, Eugenia Petrova, Alexandra Voinea, Romeo Smarandache, Mihail Ceausu

**Affiliations:** 1 Department of Pathology, “C.I. Parhon” National Institute of Endocrinology, 011863 Bucharest, Romania; dumitru_ioachim@yahoo.co.uk (D.I.); danaterzea@gmail.com (D.T.); carmen.adina83@yahoo.com (C.A.C.); mihail.ceausu@umfcd.ro (M.C.); 2 Department of Endocrinology, “C.I. Parhon” National Institute of Endocrinology, 011683 Bucharest, Romania; adinaghemi@yahoo.com (A.M.G.); jekined@yahoo.com (E.P.); alexandravoinea95@gmail.com (A.V.); 3 Department of Endocrinology, “Carol Davila” University of Medicine and Pharmacy, 020021 Bucharest, Romania; 4 Department of Radiology, “C.I. Parhon” National Institute of Endocrinology, 011863 Bucharest, Romania; anda.dumitrascu@gmail.com; 5 Department of Surgery, “C.I. Parhon” National Institute of Endocrinology, 011863 Bucharest, Romania; romeo.smarandache@gmail.com; 6 Department of Pathology, “Carol Davila” University of Medicine and Pharmacy, 022328 Bucharest, Romania

**Keywords:** Riedel thyroiditis, invasive scleroatrophic thyroiditis, obliterative vasculitis, IgG4 thyroiditis, IgG4-related disease

## Abstract

Riedel thyroiditis (RT) is a rare immune-mediated inflammatory disease that destroys the thyroid parenchyma, replacing it with storiform fibrosis extending to the extrathyroidal tissue. Secondary fibrotic lesions can be associated as parts of the systemic IgG4-related disease. We present the case of a 52-year-old female patient who presented initially with subacute thyroiditis when corticosteroid treatment was initiated. After a year, compressive respiratory symptoms and dysphagia appear, and fine-needle aspiration cytology is performed to rule out malignancy, but without results. Thyroidectomy is performed, and histopathology shows scleroatrophic thyroiditis, with chronic inflammatory infiltrate containing eosinophils extending in the neighboring tissue, rare atrophic follicles, and obliterative vasculitis. Immunohistochemistry proves abundant plasma cells with IgG4 secretion; the macrophage is mainly the M2 subtype. RT is diagnosed, and a CT (computed tomography) scan is performed to detect peritracheal fibrosis and subtle pulmonary modifications. A literature review was performed that situates our findings in the context of the current literature. The last part discusses the immuno-inflammatory mechanisms behind IgG4-related diseases.

## 1. Introduction

Riedel thyroiditis (RT) is a rare immune-mediated inflammatory IgG4-related disease (IgG4-RD) that causes chronic inflammation of the thyroid gland. It destroys thyroid parenchyma and often extends beyond the thyroid capsule, infiltrating cervical structures, vessels, nerves, and trachea, generating airway obstruction [1,2]. Its incidence is rare, with the literature citing numbers such as 1.06/100,000 or 0.06% of all thyroidectomies [3,4]. The thyroid is described as “stone-hard” or “wooden hard” due to the extensive sclerosis [2].

The pathophysiology is not clearly understood, though it seems to be of immune-mediated origin, belonging to a continuum of systemic IgG4 sclerosing diseases. It is associated with other sclerosing loci such as the orbit, mediastinum, respiratory system, and biliopancreatic tract [2,5,6,7]. Dissemination of inflammation and secondary fibrosis, known as “invasive sclerosing thyroiditis”, spreads to adjacent cervical structures, causing hypothyroidism, cervical pain, compressive respiratory symptoms, hoarseness, and dysphagia, thus strongly impacting patients’ lives [5].

It can sometimes prove to be a difficult diagnosis, as there are no specific clinical, imagistic, or laboratory instruments that can provide a precise answer, nor does it have any pathognomonic manifestations. Furthermore, association with preexistent thyroid conditions such as multinodular goiter, subacute thyroiditis, Hashimoto’s disease, or even Grave’s disease make it even more complicated to diagnose [8]. Histopathologic confirmation from open biopsy is needed for a definite diagnosis.

Treatment options are generally scarce and are often still a topic of discussion. Management includes glucocorticoids that bear a substantial risk of relapse, tamoxifen, mycophenolate mofetil, and surgery as a last resort for nonresponding disease or uncontrolled symptoms [5].

## 2. Case Report

### 2.1. Clinical Assessment

A 52-year-old white female patient presents to our Institute, as referred by a secondary center. The patient reports anterior cervical pain exacerbated by arm elevation and deep breathing, mainly in decubitus and occasional dyspnea. The patient has a 30-pack-year smoking history and no exposure to toxic conditions or radiation. On clinical examination, the patient is in good clinical condition and has a normal body weight (BMI = 24). Current thyroid examination shows an enlarged painful gland, firm to hard consistency, and a nodular mass of approximately 3–4 cm in the left lobe, mobile upon deglutition.

History from another secondary center shows that in 2022, she was diagnosed with euthyroid multinodular goiter. In 2023, echography revealed a 5 cm nodule in the left thyroid lobe, and blood tests showed euthyroidism (TSH = 0.4 μUI/mL; FT4 = 12.8 pmol/L), aTPO negative, aTG positive. In 2024, the patient presented to the same secondary center with good general condition, anterior cervical pain, low-grade fever, and biological inflammatory syndrome. Subacute thyroiditis is suspected, and the patient is started on methylprednisolone for three months with decreasing doses (32 mg-16 mg-8 mg-4 mg) and referred to our tertiary center. CT examination highlights the right thyroid lobe being within normal dimensions and a homogenous aspect, and the left lobe having two hypodense nodules of 13/7 mm and 16.5/15 mm, respectively. The latter lobe has a hyper-enhancing area of 10/4.6 mm. Upon late 2024 presentation at our center, the patient’s blood samples show mild biologic inflammatory syndrome (ESR = 25 mm/h, fibrinogen = 480 mg/dL, CRP = 2.3 mg/dL) and euthyroid function (TSH = 2.02 μUI/mL, FT4 = 18 pmol/L). After corticosteroid treatment, symptoms seem to improve clinically, with diminution of reported pain and dyspnea.

At the beginning of 2025, the patient complains of anterior cervical pain again. Blood tests show a nonspecific inflammatory syndrome (ESR = 19 mm/h, fibrinogen = 681 mg/dL, CRP = 3.18 mg/dL), thyroid markers TSH = 1.53 μUI/mL, FT4 = 16.23 pmol/L, aTPO = 41.47 UI/mL, aTG negative, and normal calcium and phosphate. The patient gained around 5 kg in weight since commencing corticosteroid medication and observed abdominal fat deposition. Current sonography highlights increased thyroid volume, a multinodular heterogenous hypoechoic structure, and a hypoechoic nodular conglomerate in the right lobe, categorized as TI-RADS 4. For the results and normal values of the blood tests, please refer to Table 1.

Fine-needle aspiration cytology (FNAC) is performed in the left lobe nodule but with unsatisfactory results (Bethesda I). Given the high TI-RADS score and the fact that corticosteroid response has diminished, symptoms appear to be exacerbated, and the patient shows signs of corticosteroid adverse effects, two months later, thyroidectomy is performed. While operating, surgeons find a pale, enlarged, and deformed thyroid gland, with extensive adhesions to the surrounding tissues including muscles, trachea, and recurrent laryngeal nerves. Complicated dissection is carried on, along with extrathyroidal adherent tissue. The surgical team did manage to preserve the laryngeal nerves. Three months after surgery, a follow-up CT examination is performed, and the blood IgG fraction profile is pre-elevated sampled. Serum IgG profiling reveals low IgG1 and 2, normal IgG3, and undetectable IgG4 (see Table 1 for values).

### 2.2. Imagistic Examination

**Figure 1:**
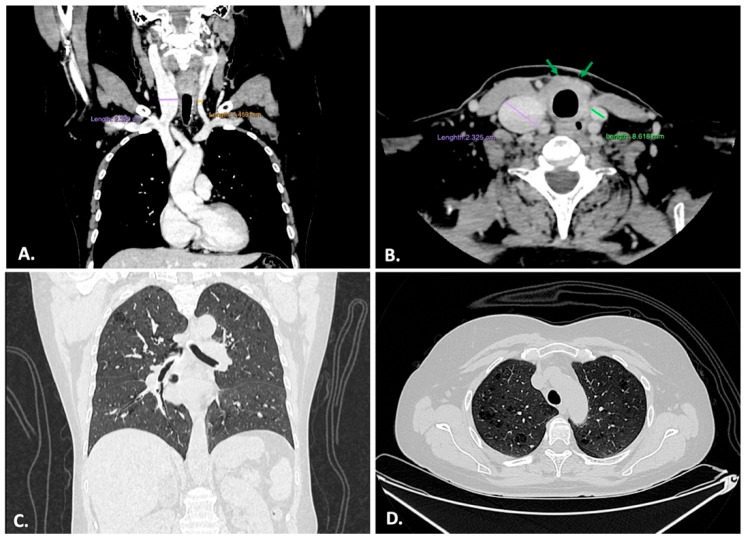
(**A**) Venous contrast showing right internal jugular vein ectasia—frontal thorax and neck. (**B**) Delayed phase showing right internal jugular vein ectasia and peritracheal fibrosis (green arrows) filling the thyroid fossa—transversal neck. (**C**) Pulmonary protocol shows subtle interstitial thickening, micronodules, rare reticular opacities, and subtle emphysematous changes—frontal thorax. (**D**) Superior lobes show the same aspects and discrete emphysematous changes—transverse thorax.

Non-contrast and contrast-enhanced CT examinations of the orbit, neck, chest, and abdomen were performed to comprehensively evaluate potential secondary fibrotic loci (see Figure 1). Venous phase contrast enhancement shows a right internal jugular vein ectasia probably due to peritracheal residual fibrosis. The entire thyroid fossa seems to be replaced by pre and peri-tracheal fibrosis (Figure 1A,B). Lung examination shows very subtle interstitial thickening, micronodules, rare reticular opacities, and subtle emphysematous changes (Figure 1C,D). No honeycombing or architectural distortions are seen, and nor are orbital or retroperitoneal changes.

### 2.3. Histopathologic Investigation

**Figure 2:**
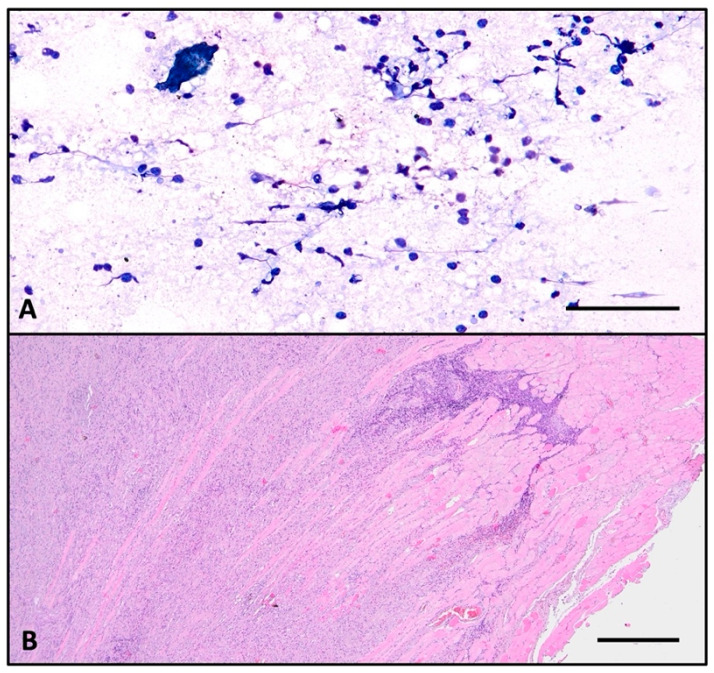
(**A**). FNAC smear stained with toluidine blue showing lymphocytes, plasma cells, very few fibroblasts, and no epithelial cells. (**B**). HE showing dense inflammatory infiltrate extending from thyroid (left) into the surrounding striated muscle (right); (**A**) scale bar—100 μm; (**B**) scale bar—500 μm.

FNAC was performed, and the sample was sent for pathological examination. The smear highlights a serous–hematic background, a loose fibrinous network, and an abundant chronic inflammatory infiltrate composed mainly of lymphocytes (Figure 2A). No epithelial cells were found, so the FNAC was labeled as non-diagnostic (Bethesda I).

Macroscopically, the total thyroidectomy specimen is tan-gray with a fibrous appearance and woody firm texture and presents with a rugged surface due to the presence of associated extrathyroidal tissue taken during excision. Sectioning reveals white-tan-colored homogenous parenchyma, no lobular pattern, and no colloid nodules, except for a 5.6 cm left lobe colloid conglomerate nodule.

**Figure 3:**
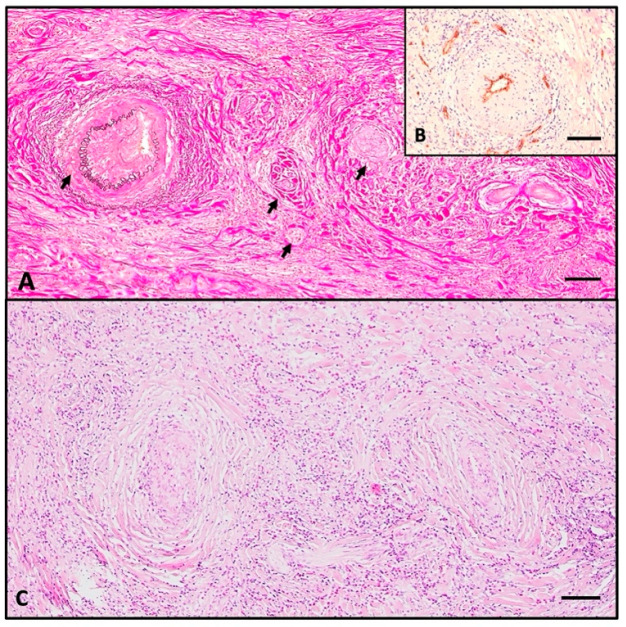
(**A**) Weigert van Gieson stain showing storiform fibrosis made of thick collagen bundles (magenta) and elastic fibers (black), delimitating internal and external lamina elastica of obliterative vasculitis-affected vessel; (**B**) CD34 showing endothelium of narrowed vessel. (**C**) HE section showing dense inflammatory infiltrate with abundant eosinophils around and within the walls of obliterated vessels. Scale bar (**A**–**C**)—100 μm.

**Figure 4:**
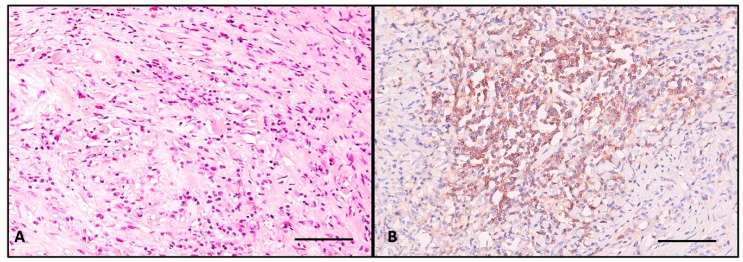
(**A**) HE stain of the dense chronic inflammatory infiltrate made of lymphocytes, plasma cells, and abundant eosinophils; (**B**) CD45 stain showing dense nonspecific inflammatory cells distributed in the entire thyroid, sometimes confluent around atrophic follicles; scale bar—100 μm.

Histologically, the normal thyroid parenchyma is replaced almost entirely by dense fibrous tissue made of thick collagen bundles and frequent fibroblasts, called storiform fibrosis (Figure 2B). Within the sclerous stroma, abundant chronic inflammatory infiltrate made of lymphocytes, plasma cells, macrophages, and frequent eosinophils is observed (Figure 3C and Figure 4). The inflammation and sclerotic process extends to extrathyroidal tissue represented by adjacent striated muscles (Figure 2B). Blood vessels have narrow lumina, with some being completely obliterated due to subintimal obliterative proliferation (Figure 3). Inside the thickened walls of both arterial and venous vessels, lymphocytes are observed. Note the internal and external lamina elastica and the lumen obliterated by a thrombus (Figure 3A,B). This manifestation is commonly named obliterative vasculitis. Near the vessels, nerve bundles are also entrapped by the constrictive fibrotic environment, with collagen fascicles forming a concentric pattern around both nerves and blood vessels.

**Figure 5:**
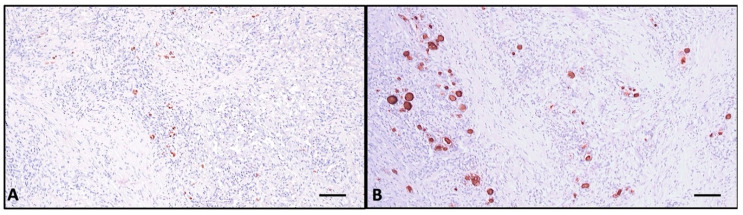
(**A**) TTF1 stain showing a few dispersed remnant follicular cells; (**B**) TG stain highlighting rare small atrophic follicles. Scale bar—100 μm.

**Figure 6:**
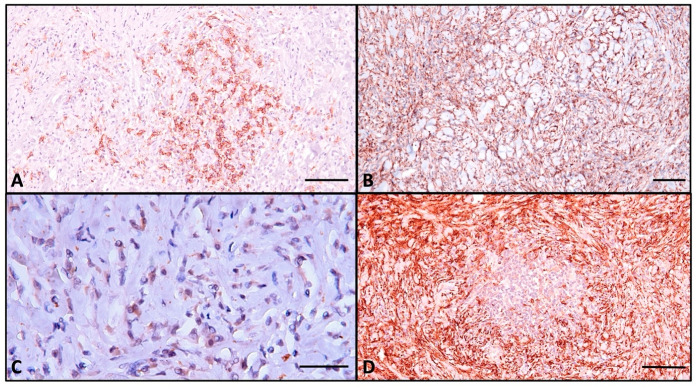
(**A**) CD138 highlights plasma cells; (**B**) CD 68 detail demonstrating the reticular network created by the macrophages in the entire thyroid; (**C**) IgG4-secreting plasma cells, >10/HPF; (**D**) CD163 shows abundant M2 macrophages, surrounding the clustered lymphoid cells around a follicular structure. Note that the majority are CD163-positive. Scale bar (**A**,**B**)—100 μm; (**C**,**D**)—50 μm.

The immunohistochemical assay identifies rare small atrophic follicles bearing TG-positive colloid, and TTF1 shows scattered follicular cells (Figure 5). CD138+ plasma cells are evenly distributed in the entire parenchyma, sometimes tending to aggregate around atrophic follicles, with most of the cells showing active IgG4 secretion (>10 cells/HPF) (Figure 6A and Figure 6C, respectively). CD68+-positive macrophages are distributed homogenously in the entire thyroid, and upon high magnification, it is apparent that they form a trabecular network with dendritic-like processes contacting each other. The majority are CD163+ macrophages, a marker of the M2 reparatory (pro-fibrotic) phenotype (Figure 6B and Figure 6D, respectively).

## 3. Discussion and Literature Review

The aim of the Discussion and Literature Review is to highlight the vast possible manifestations and clinical and paraclinical modifications, as well as to assess the high heterogeneity in the available literature.

Our report presents a patient with a short two-year history of multinodular goiter, upon which subacute thyroiditis was superimposed. Corticosteroid treatment was started; the patient did respond but only for a short period, and they rapidly began to show adverse effects. Investigations did not manage to rule out malignancy, so a total thyroidectomy was performed. Although in 2024, the secondary center suspected subacute thyroiditis and started the patient on corticosteroids, we cannot rule out that episode as the first manifestation of RT. No radioiodine uptake studies were performed at that point, and we have no access to the blood tests to rule out thyrotoxicosis. Upon presentation in our center, she had been already medicated, and symptoms were partially relieved.

RT remains an exceptionally rare diagnosis; many times, other thyroidal afflictions are better candidates according to clinical rationale [3,7,9]. It can easily be mistaken for other conditions, creating a great deal of diagnostic uncertainty. This is in part due to the fact that there are no unequivocal or pathognomonic diagnostic criteria, nor are there any definite laboratory tests that can point with certainty to this diagnosis [10]. Many cases in the literature are suspected to be malignant (anaplastic thyroid carcinoma), primary lymphoma, or even sarcoma [11]. The final answer generally comes from pathological assessment. Shafi et al. present the case of a patient who was originally diagnosed with goiter, hypoparathyroidism, and hypocalcemia; malignancy was suspected during surgery, and finally, the thyroidectomy specimen assessment turned out to be RT [10]. We aim to give a detailed pathologic report for this case and to further complement the available literature with an inflammatory immunohistochemical assay and serum immunoglobulin quantitative analysis.

FNAC is generally of no use in this diagnosis; the majority of case reports using this technique did not manage to achieve any significant results, and it even induced further confusion [10]. Moreover, sometimes, finding abundant chronic inflammatory infiltrate may lead a clinician to think that a case of primary thyroid lymphoma may be possible [10,12]. For instance, Sadacharan [13] reported a series of six patients in which preoperative FNAC was interpreted: two as Hashimoto’s thyroiditis, two suspicions of malignancy (Bethesda IV), one of unknown significance (Bethesda III), and one as indeterminate mononuclear inflammation. In our case, FNAC only showed inflammatory infiltrate and fibrin with no epithelial component and no sclerosis. This description is of almost no help, and we align with the literature by regarding FNAC as a non-diagnostic tool in RT [5,6].

The literature reports variable thyroid function in RT, but the biggest retrospective study performed on 21 patients from Mayo Clinic as well as another one from Karolinska University Hospital comprising 6 patients, revealed over 70–80% hypothyroidism [5,14]. While it is intuitive to think that advanced follicular destruction would result in hypothyroidism, on rare exceptions, the thyroid can be hyper-functional [15,16]. Given the fact that RT can develop in preexistent Grave’s disease or a toxic multinodular goiter, we cannot clearly state whether the increased function is a manifestation of the base affliction or the superimposed IgG4-mediated inflammation [15]. Nevertheless, individual case reports have highly variable results regarding thyroid function, with situations varying from hypo- to hyperthyroidism [5,8,17,18]. Our patient was euthyroid until thyroidectomy. The explanation may reside in the fact that she had retained a small number of atrophic follicles, which produced enough hormones to achieve homeostasis. For a tabular form of the data reported in the literature, see Table 2.

McIver et al. [19] presented a case of RT successfully treated with Tamoxifen and corticosteroids upon initial presentation. Four years later, the patient returned for a programmed checkup when blood, iodine uptake, and imagistic tests suggested Grave’s disease in the nonfibrotic thyroid rest.

A considerable number of case reports correlate RT presentations with primary hypoparathyroidism, probably due to fibrotic infiltration of the neighboring gland [10,20]. Salhi et al. [20] show a complete resolution of hypoparathyroidism upon corticosteroid administration and thyroidectomy with parathyroid preservation. Shafi et al. [10] present the case of a 35-year-old man who underwent left lobectomy, postoperatively treated unsuccessfully with Tamoxifen and Rituximab, and was later moved onto corticosteroids, without hypoparathyroidism resolution.

Thyroid autoantibodies are yet again a confusion-inducing variable. The literature cites a highly variable prevalence for aTG and aTPO, and it is of no surprise since, in many cases, RT is associated with preexistent conditions. Falhammar et al. showed, in their patient population, three patients being aTPO-negative and two being aTPO-positive, whereas two were aTG-positive and two were negative [5]. Fatourechi et al. [14] state that 9 out of 10 patients had at least one positive antibody. We therefore conclude that the available data does not converge to a unanimous criterion; moreover, antithyroid antibodies are, in the case of RT, of no known significance. In their review, Carsote et al. find equal numbers of thyroid autoantibody-positive and -negative cases reported in the literature [3,10,21,22,23]. Interestingly, in 2023, our patient was aTG-positive and aTPO-negative, as per the secondary center, whereas in 2025, the profile was opposite: aTG-negative and aTPO-positive. Anti-TSH receptor antibodies were negative throughout.

Blood tests in RT generally show modified inflammatory markers, suggesting biological inflammatory syndrome. Fatourechi et al. [14] computed the mean value of ESR for their 21-patient cohort, and it was found to be within normal limits. The same conclusion was reached by Canpolat et al. [24] in their eight-patient group, where only one patient presented an elevated level, whereas eight out of nine had increased CRP. Falhammar et al. [5] identified CRP above normal limits in five out of six patients, with some even reaching values as high as 10 times the normal value. Many other independent case reports show increased CRP value [3,10,20,22,25]. In any case, generally speaking, CRP tends to show elevated blood values, as the majority of studies suggest [3,10,20,23]. There are scarce writings that cite elevated white blood cell count in blood samples [4,11,25]. Our patient had normal ESR but mildly elevated CRP and fibrinogen.

Serum IgG4 levels alone, or in the form of IgG/IgG4, have been postulated as important tools for diagnosis. Although many authors collected blood samples from their patients, there is almost no significant correlation between immunoglobulin serum levels and the presence of RT [26]. For instance, Canpolat et al. [24] shows normal serum levels of both IgG and IgG4 in all of his eight patients, whereas Jin [27] reports increased serum IgG4 in two out of five patients. Individual case reports show high levels of serum IgG4 [28,29]. Our patient had low IgG and IgG2 and undetectable IgG4. Low IgG2 correlates with chronic sinusitis, as confirmed by CT examination.

Our case report is inherently subjected to limitations, firstly, due to the fact that we only report the case of a single patient. One limitation is that we did not have access to the complete medical history from the first center where the patient was treated. Secondly, IgG fractions were only measured after histopathological confirmation, as we did not expect a case of RT, given its very rare incidence. As we see it, very few writings report IgG fractions before surgery. When interpreting the values of IgG1 and 2, the presence of the rheumatoid factor, immune circulating complexes, or paraproteins was not assessed to exclude analytical interferences. IgG4 remains to be checked in the further follow-up consults, in correlation with clinical evolution. Also, the widely variable numbers in the literature could be explained by the method used for IgG profiling: nephelometry or turbidimetry.

Secondary fibrotic loci can sometimes accompany thyroidal expression, since RT is part of a bigger IgG4 systemic multifocal fibroinflammatory syndrome [30]. Jakobiec et al. [31] presented the case of a patient with metachronous RT, lacrimal gland involvement with swelling, and a solitary apical pulmonary nodule. All sites were biopsied, and numerous lymphocytes, plasma cells, and IgG4-producing plasma cells were present, although fibrosis was noted only thyroidally and pulmonary. Nielsen et al. [25] reported the case of a 59-year-old female who presented with low-grade fever, a hard, painful goiter, bilateral orbital involvement, elevated CRP, and hypothyroidism. To exclude malignancy, a wedge biopsy was taken, which showed extensive fibrosis. RT with orbital involvement as the first clinical manifestation was the definitive diagnosis. Sadacharan [13], on the other hand, reported no secondary involvement in his six-patient series. In a larger systematic review from 1988, Schwaegerle et al. [16] reported rates of 34% of RT associated with other fibrotic manifestations. In our case, peritracheal fibrosis and only subtle pulmonary changes were detected, but no orbital involvement or retroperitoneal fibrosis was evident.

Concerning the histopathological and immunohistochemical aspects, we found that the majority of the gland showed storiform fibrosis. IgG4 staining was performed to prove, together with CD138, that within the fibrous stroma, plasma cells were secreting this immunoglobulin and thus the affliction belonged to IgG4-RD. Not only do the majority of patients with RT have positive immunohistochemistry for IgG4 within the destroyed glandular parenchyma, they also have an increased IgG4/IgG ratio [4,29]. Canpolat [24] shows that 2/8 patients have an increased ratio, whereas Yu [32] claims 3/5. Stan et al. [6] targeted three patients with other IgG4-related complications and managed to histologically sample both the thyroid and the secondary affected tissue, proving that both had increased counts of IgG4-secreting plasma cells.

**Table 2:** Table synthesizing data extracted from case reports from the literature.

First Author	Year	n	Thyroid Function at Presentation	Inflammation	Antithyroid Antibodies	Serum IgG	IgG/IgG4 Tissue > 40%	Main Manifestations	Secondary Fibrosis Involvement	Tobacco Use	Associated Thyroid Condition
Pandev [3]	2023	1	− hypothyroidism	1 ESR ↑, CRP ↑ (1)	aTP ↑, aTG ↑	NA	NA	− enlarging neck mass − dysphagia − dyspnea	NA	NA	Hashimoto’s thyroiditis
Falhammar [5]	2018	6	− 1 normal − 5 hypothyroidism − 1 hypoparathyroidism	ESR ↑, CRP ↑ (1)	NA	IgG4 ↑ (1) unknown (2)	yes (1)	− dyspnea − cough − tiredness	yes (1)	NA	NA
Won [8]	2006	1	NA	NA	NA	NA	NA	NA	NA	NA	goiter
Shafi [10]	2020	1	hypothyroidism	NA	aTP ↑ aTG ↑	normal	NA	− enlarging neck mass − dysphagia − dyspnea	no	yes	goiter
Sadacharan [13]	2022	6	− normal (5) − hypothyroidism (1)	NA	aTP ↑ aTG ↑	NA	NA	− painful neck mass − dyspnea − dysphagia	no	NA	NA
Fatourechi [14]	2011	21	− normal (5) − hypothyroidism (14) − hypoparathyroidism (3)	NA	NA	NA	NA	− painful neck mass − dysphagia	yes (8)	yes (16)	Grave’s disease (3)
Lee [15]	2013	1	hyperthyroidism	ESR N CRP N	aTSH-R ↑	NA	NA	− painless neck mass − hoarseness	yes	NA	Grave’s disease
Schwagerle [16]	1988	1	NA	NA	NA	NA	NA	− painless neck mass	no	NA	NA
Boakye [17]	2025	1	hypothyroidism	ESR N CRP N	aTP ↑, aTG ↑	normal	NA	enlarging neck mass	no	yes	hypothyroidism
Ezanolue [18]	2021	1	normal	NA	NA	NA	NA	− enlarging painful neck mass − dyspnea	no	no	euthyroid goiter
McIver [19]	2010	1	normal	NA	aTSH-R ↑	NA	NA	− painful neck mass − dyspnea	no	NA	Grave’s disease
Salhi [20]	2023	1	− hypothyroidism − hypoparathyroidism	CRP ↑	NA	NA	NA	− dysphagia − dyspnea	NA	no	goiter
Navarro-Sánchez [22]	2020	1	− hypothyroidism	ESR ↑	aTP ↑, aTG ↑	IgG1 N IgG2 ↑ IgG3 N IgG4 ↑	40%	− enlarging neck mass − dysphagia	no	no	hypothyroidism
Góralska [23]	2021	1	− hypothyroidism	ESR ↑ CRP ↑	aTP ↑, aTG ↑	IgG4 ↑	NA	− enlarging painful neck mass − dysphagia − hoarseness	no	NA	nodular goiter
Canpolat [24]	2020	8	− 1 hypoparathyroidism	ESR ↑ (4) CRP ↑ (7)	aTP ↑, aTG ↑ (3) aTSH-R ↑	IgG N (8) IgG4 N (8)	yes (2)	− enlarging neck mass − dyspnea	yes (3)	yes (3)	NA
Nielsen [25]	2003	1	− hypothyroidism	ESR ↑ CRP ↑	aTP ↑ aTSH-R N	NA	NA	− enlarging painful neck mass − fever	yes	NA	NA
Wang [4]	2012	1	− normal	ESR ↑ CRP ↑	aTP ↑, aTG ↑ aTSH-R N	NA	NA	− neck pain	NA	NA	Hashimoto’s thyroiditis
Pusztaszeri [26]	2012	1	− hyperthyroidism	NA	aTP ↑, aTG ↑	IgG ↑ IgG4 N	no	− enlarging painful neck mass	no	no	goiter
Jin [27]	2022	2	− hypothyroidism	NA	aTP ↑ (1) aTG ↑ (2)	IgG4 ↑ (1)	yes (1)	− enlarging neck mass − dysphagia	NA	NA	NA
Amaroui [28]	2013	1	− normal	NA	aTP N, aTG N	IgG ↑ IgG4 ↑	NA	− none	retroperitoneum	no	no
Takahashi [29]	2023	1	− hypothyroidism	NA	aTG ↑ aTSH-R ↑	IgG N IgG4 ↑	yes	− enlarging neck mass	NA	yes	Grave’s disease
Yu [32]	2021	5	NA	NA	NA	NA	yes (3) no (2)	NA	NA	NA	NA

NA, not assessed; aTG, anti-thyroglobulin antibody; aTP, anti-thyroperoxidase antibody; aTSH-R, antithyroid-stimulating hormone receptor antibody; CRP, C-reactive protein; ESR, erythrocyte sedimentation rate, ↑ increased levels, N normal levels.

**Figure 7:**
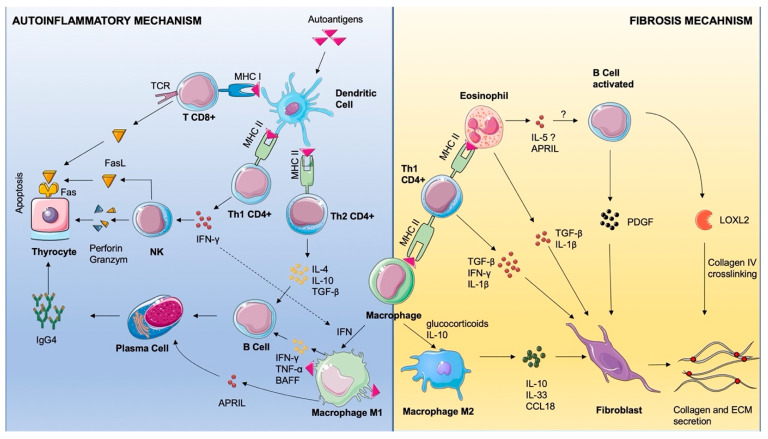
Schematic representation of the possible mechanism of Riedel Thyroiditis. Dendritic cells uptake the cellular debris that is presented to T CD8+ via MHC I and to Th1 CD4+ and Th2 CD4+ via MHC II. Th1 secretes IFN-γ that activates NKs and polarizes macrophages to the M2 phenotype. NKs induce apoptosis of the thyrocyte by perforin–granzyme attack or through FasL–FasR interaction. CD8+ induce apoptosis through the same FasL–FasR interaction mediated by TCR. Th2 activate B cells through IL-4, IL-10, and TGF-β, and M1 activates B cells through IFN-γ, Tumor Necrosis Factor Alpha (TNF-α), and B Cell Activating Factor (BAFF). Plasma cells are activated by APRIL and secrete IgG4 against the thyrocyte.Fibrosis is sustained by M2 macrophage polarized by glucocorticoids and IL-10, activating the fibroblast by IL-10, IL-33, and chemokine C-C motif ligand 18 (CCL-18). Th1 activated by macrophage or eosinophil presentation via MHC II secretes TGF-β, IFN-γ, and IL-1β, activating the fibroblast. Eosinophils activate fibroblasts by TGF-β and IL-1β secretion and they may activate B cells through IL-5 and APRIL, through an unclear mechanism. B cells activate fibroblasts by PDGF and contribute to fibrosis by secreting lysyl oxidase-like 2 (LOXL2) enzyme that crosslinks collagen fibers. The image is adapted from Servier Medical Art (https://smart.servier.com/), licensed under CC BY 4.0 (https://creativecommons.org/licenses/by/4.0/).

Although cellular and humoral mechanisms of IgG4-RD are incompletely understood, let alone the particular form of RT, some data is available in the literature. We will now focus on the pathophysiology and immunology of these events (Figure 7). Under numerous environmental and genetic factors, central and peripheral immunological tolerance can be disrupted. Upon this disruption, the immune system targets self-structures and starts the inflammatory cascades [33]. Cellular debris is engulfed by professional antigen-presenting cells (APCs)—dendritic cells, macrophages, and B cells—and it is further presented to CD4+ lymphocytes through major histocompatibility complex (MHC) class II molecules [34]. Many other unprofessional antigen-presenting cells can do the same through MHC class I and subsequently activate CD8+ T cells [34]. Once activated, CD8+ induces apoptosis of the targeted cell by T cell receptor-dependent activation of the Fas Receptor (FasR)–Fas Ligand (FasL) signaling pathway [35]. Professional APCs also present antigens through MHC II both to CD4+ Th1 and CD4+ Th 2 [36]. In the IgG4-RD context, the same activation can be achieved by eosinophils presenting antigens, interestingly through MHCII [37]. CD4+ Th1 secrete vast amounts of Interferon-γ (IFN-γ) that will subsequently activate Natural Killer cells (NKs) that secrete Perforins, which create pores in the targeted cell membrane through which Granzymes enter the cytoplasm [36]. Granzyme B, a serine protease, cleaves Caspase-3 and induces apoptosis [36]. CD8+ cells can use the same Perforin and Granzyme mechanism [35,36]. IFN-γ has many other proinflammatory roles, including M1 macrophage polarization and increased antigen presentation, Th1 differentiation, B cell proliferation and IgG class switching, and increased CD8+ toxicity [38].

Upon activation, CD4+ Th2 secretes TNF-α, IL-4, and IL-10 that activate B cells and subsequent polarization in plasma cells [36]. A switch in immunoglobulin production happens, and the cell becomes autoreactive, secreting IgG4 against the thyrocyte.

For a better understanding of the autoinflammatory and fibrotic mechanism, we chose to use antibodies to characterize the macrophage infiltrate. CD68+ cells are homogenously distributed, forming a reticular network made of interconnected cell processes. The majority of macrophages are also positive for CD163, showing that they belong to the M2 phenotype [39]. To our knowledge, no other RT case report has addressed the macrophage population through immunohistochemistry [40]. Macrophages promote autoinflammation through B cell activation with TNF-α, IFN-γ, and BAFF. Also, macrophages secrete A proliferation-induced ligand (APRIL) that promotes plasma cell survival [40].

The CD163 molecule is used as a reliable marker for M2 polarization in cells that play important roles in fibrosis mechanisms [41]. M2 polarization can be achieved by administering corticosteroids and directly by IgG4 [42,43]. In vitro, monocytes treated with glucocorticoids decrease the secretion of proinflammatory cytokines, as well as increasing the secretion of anti-inflammatory cytokines [42,44]. Given the fact that our patient was administered glucocorticoids for a period of three months, the majority M2 phenotype present here could be an expression of the therapeutic effect. M2 macrophages secrete IL-10, IL-33, and CCL-18, which directly induce collagen and extracellular matrix by the fibroblast and induce tissue repair [45,46]. CD4+ Th1 cells secrete TGF-β and IL-1β, which activate fibroblast collagen secretion [47]. B cells isolated from patients with IgG4-RD secrete PDGF-β, which increases collagen and enzymes implicated in extracellular matrix remodeling [48]. Also, they participate in IgG4-related fibrosis by secreting LOXL2, an enzyme that crosslinks collagen and elastin fibers [48].

In all IgG4-related diseases, eosinophils are an important part of the inflammatory infiltrate. It seems that these cells can function as APCs, as they can express MHC II upon granulocyte-macrophage colony-stimulating factor (GM-CSF) stimulation and they are able to present antigens to T CD4+ [37]. They also participate in sustaining autoimmunity by activating B cells through IL-5 secretion and plasma cells through APRIL [47,49]. Eosinophils promote fibrosis, probably through fibroblast activation and fibronectin deposition by IL-1β and TGF-β. Further studies on cellular and humoral inflammatory response in the case of RT might pave the way for a better understanding and better treatment options for this condition. A larger series of cases should be assembled, with the aim of achieving the homogenous testing and reporting of immunohistochemical markers and serologic characterization both in terms of IgG and blood cytokine profiling. To achieve further insights into pathophysiology, we need fundamental research such as peripheral blood characterization of immune populations, as well as molecular insights into the transcriptomics and gene expression that target inflammatory regulation, fibrotic response, and pathway identification.

## 4. Conclusions

Riedel thyroiditis is a rare IgG4 fibrosing thyroiditis that is sometimes associated with other coexisting fibrosing lesions. Although there are no specific clinical signs, imagistic data, or laboratory markers, it should be suspected when the patient presents with a firm painful anterior cervical mass. Histopathologic confirmation from open biopsy is needed for a definite diagnosis. Storiform fibrosis, follicular atrophy, massive infiltration of thyroid capsule, concentric perivascular and perineural sclerosis and inflammation, obliterative vasculitis, an increased number of eosinophils, plasma cells, especially IgG4-type, and macrophages are constituents of the histopathological picture of RT. Histopathologic examination yields a definitive answer and should always be performed either in open biopsy or on a thyroidectomy specimen.

## Figures and Tables

**Table 1:** Patient blood test values and normal range.

Year	Laboratory Test	Patient Values	Normal Range
**2023**	**TSH**	0.4 μUI/mL	0.35–4.94
	**FT4**	12.8 pmol/L	12–22
	**aTPO**	negative	negative *
	**aTG**	positive	negative *
**2024**	**ESR**	25 mm/h	2–25
	**fibrinogen**	480 mg/dL	200–400
	**CRP**	2.3 mg/dL	0–0.5
	**TSH**	2.02 μUI/mL	0.35–4.94
	**FT4**	18 pmol/L	12–22
**2025** **before surgery**	**ESR**	19 mm/h	2–25
	**fibrinogen**	681 mg/dL	200–400
	**CRP**	3.18 mg/dL	0–0.5
	**TSH**	1.53 μUI/mL	0.35–4.94
	**FT4**	16.23 pmol/L	12–22
	**aTPO**	41.47 UI/mL	0–5.6
	**aTG**	55.26 UI/mL	0–115
**2025** **after surgery**	**IgG1**	3.9 g/L	4.05–10.11
	**IgG2**	1.24 g/L	1.69–7.86
	**IgG3**	0.32 g/L	0.11–0.85
	**IgG4**	0.00 g/L	0.03–2.01

* qualitative report, as provided by the secondary center.

## Data Availability

The materials and methods used in this study are available as Supplementary Materials.

## Supplementary Materials

The supporting information can be downloaded at: https://www.mdpi.com/article/10.3390/ijms26167786/s1.

## List of Abbreviations

APRIL	A Proliferation-Inducing LigandaTG-anti-thyroglobulin antibody
aTPO	antithyroid peroxidase antibody
BMI	body mass index
CCL	chemokine C-C motif ligand
CRP	C-reactive protein
ESR	erythrocyte sedimentation rate
FasL	Fas ligand
FasR	Fas receptor
FNAC	Fine-needle aspiration cytology
FT4	free T4
HPF	high-power field
IFN-γ	Interferon Gamma
IgG4-RD	Immunoglobulin 4-related disease
IL	interleukin
LOXL2	Lysyl oxidase-like 2
MHC	Major Histocompatibility Complex
PDGF	Platelet-derived growth factor
RT	Riedel thyroiditis
TCR	T cell r
TGF-β	Transforming Growth Factor Beta
TI-RADS	Thyroid Imaging Report and Data System
TNF-α	Tumor Necrosis Factor Alpha
TSH	thyroid-stimulating hormone
