# Supplementary material for: IgG4-Mediated Sclerosing Riedel Thyroiditis: A Multidisciplinary Case Study and Literature Review
Source: Int J Mol Sci. 2025 Aug 12;26(16):7786. doi: 10.3390/ijms26167786 (PMC12386525; doi:10.3390/ijms26167786)
Supplement: Supplementary file 1 [file ijms-26-07786-s001.zip › ijms-3714127-supplementary.pdf]

Supplemental Document

Material and Method

The total thyroidectomy specimen was fixed in 10% neutral buffered formalin for 24 hours. During macroscopic handling, representative tissue samples were collected and further processed to obtain paraffin-embedded tissue blocks. Histologic sections were cut at 3um thickness. Several sections were stained with standard hematoxylin-eosin, using Tissue-Tek® DRS™ 2000 automated stainer (Sakura Finetek Europe, Netherlands). Weigert Van Gieson special stain (Bio-Optica, Italy) was done according to the manufacturer's protocol. Immunohistochemistry was performed with Leica Bond Max Automated IHC and ISH Staining System (Leica Biosystems, United States), using antibodies listed in Table 1. Detection was carried on with BOND Polymer Refine Detection System (Leica Biosystems, United States).

Representative photomicrographs were captured using Olympus BX50 transmitted light microscope paired with Olympus DP28 camera. For image acquisition and analysis, OLYMPUS cellSens Standard 4.1 was used.

Antibody Target	Target Structure	Producer	Cat #
CD 34	Endothelium	Leica Biosystems, UK	PA0212
CD 45	Leucocytes	Leica Biosystems, UK	NCL-LCA
CD 68	Macrophages	Zeta Corporation, USA	Z2732
CD 138	Activate lymphocytes	Zeta Corporation, USA	Z2490
CD 163	M2 Macrophages	Zeta Corporation, USA	Z2782
IgG 4	IgG4 secreting plasma cells	Zeta Corporation, USA	Z2366
TG	Thyroglobulin, colloid	Leica Biosystems, UK	NCL-L-THY
TTF1	Follicular cells	Leica Biosystems, UK	PA0364
